# Snow-dirt sludge as an indicator of environmental and sedimentation processes in the urban environment

**DOI:** 10.1038/s41598-019-53793-z

**Published:** 2019-11-21

**Authors:** Andrian Seleznev, Ilia Yarmoshenko, Georgy Malinovsky, Ekaterina Ilgasheva, Elena Baglaeva, Anastasia Ryanskaya, Daria Kiseleva, Tamara Gulyaeva

**Affiliations:** 1 Institute of Industrial Ecology, Ural Branch of Russian Academy of Sciences, Postal address: S. Kovalevskaya Str., 20, 620990 Ekaterinburg, Russia; 2 The Zavaritsky Institute of Geology and Geochemistry, Ural Branch of Russian Academy of Sciences, Akademika Vonsovskogo Str., 15, 620016 Ekaterinburg, Russia

**Keywords:** Environmental monitoring, Geochemistry, Pollution remediation, Environmental impact, Environmental impact

## Abstract

The formation of snow-dirt sludge (SDS) via the mixing of snow and surface-deposited sediment by vehicles and pedestrians is one of the least studied sedimentation processes in urban areas. The aim of this study was to analyse the material, mineral, particle size, and chemical compositions of the SDS solid phase. The study was conducted using Ekaterinburg, Russia, as an example city with an intense anthropogenic impact and a long cold snowy period. The average content of the SDS solid phase was approximately 35 g L^−1^ of meltwater on heavy traffic roads, of which the dust fraction (<0.1 mm) accounted for 23 g L^−1^. On driveways and lawns, the contents of the SDS solid phase were 12 and 6.6 g L^−1^, respectively. The mineral composition of the SDS solid phase was generally similar to the geology of the surrounding area, which is composed of a mosaicked combination of felsic and mafic complexes. The presence of building material fragments and other anthropogenic particles confirms the significant anthropogenic impact. The chemical composition contained major and minor elements specific to the minerals and materials that constitute the SDS solid phase. There was significant variability in the concentrations of Pb, Cu, and Zn in the granulometric fractions, indicating pollution. Intensive melting of SDS with a high particulate matter (PM) content is an important factor influencing the environmental quality of the urban environment.

## Introduction

Contemporary sedimentation processes play a significant role in shaping urban environmental quality. In addition to natural factors, such as weathering and erosion, there is an intense anthropogenic impact on exposed surfaces in urban areas. As a result, the typical yield of deposited material in cities exceeds the rate of this process in natural and agricultural landscapes by an order of magnitude^1,2^. An urban sediment cascade model recognizes the relationship between sediment sources and downstream sedimentation^3,4^. Intermediate stages of the cascade include transport mechanisms, paths of migration, deposition on various urban surfaces and in gullies, and removal via storm sewers. Deposition on roads is commonly considered the most significant sedimentation process in the urban environment^3–5^. In a broader sense, sediment deposited on various urban surfaces, such as roads, sidewalks, driveways, and playgrounds, is referred here as urban surface deposited sediment (USDS) and is considered a particular compartment of the urban environment.

The intermediate stages of the sediment cascade are associated with the deposition and storage of USDS, and they reduce the environmental quality and contribute to unfavourable perceptions of the urban environment. Significant deposits with thicknesses greater than 5 cm are observed in local depressions in the microrelief^6,7^. USDS contains a significant amount of fine particulate matter (PM), including the highly transportable PM_2.5_ and PM_10_ fractions, which, if dispersed in the atmosphere, pose significant health risks^4,8–11^. USDS is characterized as a non-point source pollutant with organic and inorganic toxicants, including pathogens, in a city^12^. Many authors have noted elevated concentrations of potentially harmful elements (PHE) in road-deposited sediment^13,14^. Chemical and granulometric analysis of USDS, in particular road-deposited sediment, is included in several cities’ environmental monitoring programmes^15,16^.

Rainwater and meltwater runoff represent a significant stage in the sediment cascade because they capture and transport USDS into depressed parts of the landscape and storm sewers. Urban runoff depends on site-specific characteristics such as land use, the layout of relief, and other factors that are anthropogenic in nature^17–20^. Lateral sediment transport driven by water runoff is enhanced on the impervious surfaces common in the urban environment^19^.

Seasonality is an important factor determining runoff characteristics. In regions with long snowy winters, an increase in surface precipitation runoff occurs during the springtime melt^17,18,21^. The solid matter suspended in the runoff water varies in mass, concentration, grain size, and pollutant content during cold and warm seasons^22–24^. The sediment formation process in winter in cities is enhanced by enhanced road wear due to the use of studded tyres^25–27^. Winter combustion of biomass and fossil fuels supply large quantities of soot particles to the USDS^28,29^. Anti-icing agents on roads and sidewalks also become part of the road- and sidewalk-deposited sediment^22^. Snow itself contains PM of various origins^30^, and deicing agents change the salinity and pH of snow^22^. Thus, unlike other seasons of the year, runoff contains high concentrations of pollutants and solid sediment during the short period of snow melt in spring^23,31^.

In urban landscapes, snow cover inevitably interacts with USDS and modifies the depositional process. Due to its porous structure, snow can accumulate and store a large quantity of pollutants and road-wear products^32^. Snowbanks along urban highways act as passive sinks for both metal elements and solids generated by traffic and maintenance activities^32,33^. Some authors have noted that there is little known about the role of frozen precipitation in contemporary sedimentation processes in urban environments in regions with cold winters and long periods of steady snow cover^20,21,32^. One of the least studied objects in sedimentogenesis under winter conditions in the urban environment is snow-dirt sludge (SDS) (or snow-sediment sludge). This material is formed as a result of the mixing of snow and USDS through the actions of vehicle wheels and pedestrian feet. In the process of winter cleaning and snowplough activities, SDS is moved from roads, sidewalks, and driveways to roadsides and lawns. Furthermore, SDS is partially transported to special landfills and other storage sites.

The formation of dirty snow piles is often negatively perceived by people, as is reflected in media coverage. Examples of such publications are easily found in media from the USA^34,35^, Canada^36^, Norway^37^, Sweden^38^, South Korea^39^, and Russia^40^.

There is a general understanding regarding the causes of SDS formation and recognition of SDS toxicity, although scientific data obtained from systematic observations and analysis of the factors influencing the formation and the composition of SDS are lacking. The aim of this study was to analyse the material, mineral, particle size, and chemical compositions of the SDS solid phase. The study was conducted using Ekaterinburg as an example city with a large population, an intense anthropogenic impact, and a long cold snowy period.

## Materials and Methods

### General description of Ekaterinburg

**Figure 1:**
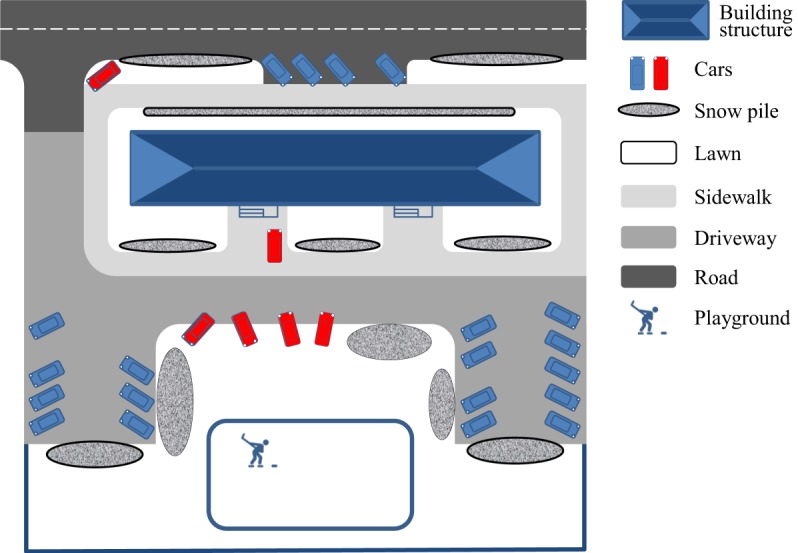
The generalized layout of a typical sampling site.

Ekaterinburg lies in the eastern foothills of the Middle Urals (56°50′N, 60°35′E; see Fig. 1S (Supplementary information)). The total area of the city and the area encompassing the city’s residential districts are 1,143 km^2^ and 128 km^2^, respectively. A significant part of Ekaterinburg is located within the watershed of the Iset River. The relief of the city consists of floodplain terraces along the Iset River and hilly plains. Ekaterinburg is the fourth largest city in Russia, with a population of approximately 1.5 × 10^6^. While a number of industries have developed in the city since its foundation and Soviet industrialization, a complex of scientific, technical, and design organizations now dominate the city’s economy. The city’s electricity supply is mainly based on power plants that switched from burning coal to natural gas from eastern Siberia in the 1980s–90s, with some contribution from a nuclear power plant. The number of road vehicles in the city has tripled since 1990, reaching approximately 620,000 in 2017.

The climate in the Middle Urals is temperate continental with prevailing western transport of air masses. The long-term average (for the period from 1881 to 1980) January and July temperatures are −15.5 and +17.2 °C, respectively^41^. Although the summer temperature is similar to the average for European cities, due to the low winter temperature, Ekaterinburg is one of the coldest cities with a population of over a million people in Europe and Russia.

### Meteorological conditions of the study period

The SDS sampling was conducted in the period from February 21 to March 19, 2017. The 2016–2017 winter period was meteorologically characterized as follows. In the fall of 2016, the transition to an average daily air temperature below 0 °С was first observed on October 15, and a steady transition occurred on October 26, which is 6 days later than the average date determined from long-term observations^41^. On February 21, 2017, the average daily air temperature exceeded 0 °С for the first time during a three-day thaw. A steady increase in the average daily air temperature above 0 °С occurred on April 6, 2017, which matches the average based on long-term observations.

In the fall of 2016, the first temporary snow cover was recorded on October 13, and steady snow cover formed on October 30 (7 days earlier than the average date). The largest average snow depth was noted in February 2017 at 47.9 cm, which is 11.6 cm above the norm (according to the public available weather archive, www.rp5.ru). By the end of winter in March 2017, the average snow depth was 35.1 cm, which is 1.8 cm above the norm. During the cold period, 138 mm of precipitation accumulated, which exceeds the norm by 24 mm, and evaporation from the snow surface was 34.2 mm. Thus, for the 2016–2017 cold period, 103.8 mm of water was accumulated in the snow cover.

The steady snow cover lasted 163 days, which is 10 days longer than the long-term average, and collapsed on April 7, 2017. Intensive day-and-night snow melting lasted 9 days from March 4 to 7, from March 25 to 27, on March 29, and on April 6. The daytime snowmelt lasted 32 days from March 2 to 30 and from April 3 to 5.

### Study design

Six sampling sites representing typical quarters of the city were selected in Ekaterinburg. The selected blocks were randomly chosen from the microrayons (microdistricts) constructed in different periods and located in different geographical parts of the city. Main characteristics of the sampling sites are represented in Table S1 in Supplementary information. The sampling sites adjoined streets with different traffic intensities.

A landscape survey was conducted at each sampling site. During the survey, an urban landscape specialist made a site description according to a special questionnaire adapted for the study of conditions influencing solid sedimentary material formation and transport.

**Figure 2:**
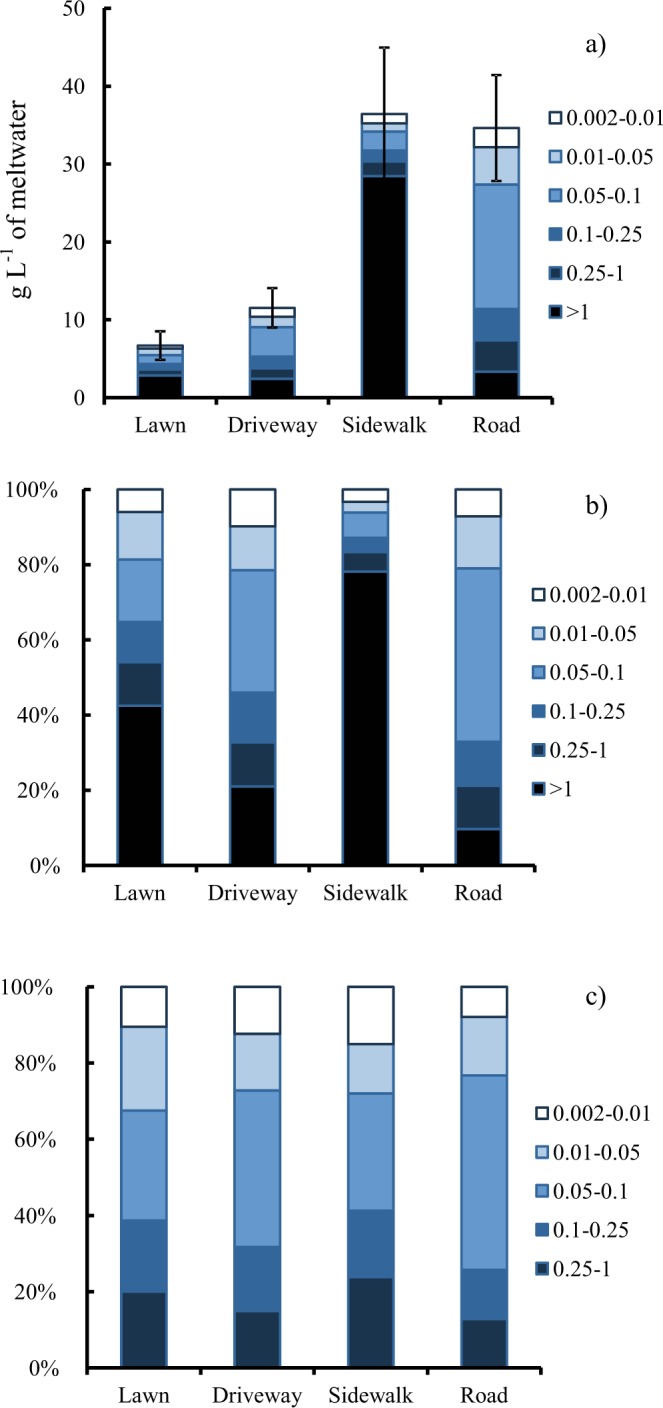
The results of the particle size composition analysis of solid phase material in the SDS (mm). (**a**) Absolute content of solid material in meltwater (g L^−1^) with standard deviation; (**b**) relative contribution of size fractions to the mass of solid material in SDS; (**c**) relative contribution of size fractions to the mass of solid material in SDS without fraction of >1 mm.

Snow and SDS samples were taken at each site according to the following scheme. Samples of snow were collected from landscaped areas with undisturbed snow cover (lawns inside and outside the block and playgrounds in courtyards) with a 10 cm diameter plastic sampling tube according to the Manual for the Control of Pollution of the Atmosphere RD 52.04.186-89. Five snow cores of the full depth of the snow cover were collected in the given landscape area and then unified into one sample. Particles of soil, grass and small debris at the bottom of the sampling core were removed. The samples of SDS were collected from roads and roadsides, sidewalks, driveways, parking lots, and piles of snow inside and outside the residential block. SDS samples were taken with a steel shovel. Each SDS sample was combined with material collected by five scoops within the given landscape area. The sample volume of the unified sample of undisturbed snow cover and the SDS sample taken in the given landscape area was 12 litres. The shovel and snow sampling tube were cleaned between samples with a dry wipe or brush. The landscape area over which the unified sample of undisturbed snow cover and the SDS sample were collected was at least 10 m^2^. Examples of snow and SDS sampling are presented in Figs. 2S–7S in Supplementary information. Number of undisturbed snow and SDS samples are represented in Table S2 in Supplementary information.

### Landscape survey of sites

The landscape survey included visiting the sampling site, visually inspecting the site to delineate microlandscape functional zones, and describing the layout and characteristics of the areas in the outlined zones. The list of characteristics within microlandscape functional zones included type (e.g., road, sidewalk, driveway, lawn, or playground); surface type (e.g., asphalt, gravel, or lawn grass), its technical conditions, and characteristics of snow cover; general technical condition; availability and type of parking (organized or unorganized); the number of parking lots; the number of cars parked at the time of the survey; snow removal characteristics; the presence of earth works and the type of works; and the presence of solid sediment sources.

### Granulometric analysis

A granulometric analysis was carried out on each collected sample. The samples of snow and SDS were melted at ambient temperature in the laboratory. Leaf and plant fragments were removed from the melted sample. The volume of water equivalent to melted snow pack (the total amount of liquid and solid material) was measured. The decantation procedure was conducted multiple times according to the procedure described in Test Method WA 115.1–2017^42^ to separate the solutions with particles sized 0.002–0.01 and 0.01–0.05 mm. The length of time the liquid was allowed to stand was calculated based on Stokes’ Law to obtain particle size fractions 0.002–0.01 mm and 0.01–0.05 mm, such that after the elapsed time, only particles with the desired size will be in suspension. The solid material with particle size fractions of 0.002–0.01 mm and 0.01–0.05 mm was acquired by vacuum filtering of the solutions through cellulose filters with pore sizes of 0.002 mm and 0.008 mm, respectively.

To obtain the fraction with particle sizes of 0.05–0.1 mm, the remaining portion of the sample was sieved through a 0.1 mm sieve with the addition of distilled water. Afterward, the remaining sample was sieved through a 0.25 mm sieve with the addition of distilled water to separate the particle size fraction 0.1–0.25 mm. The remaining material was dried and sieved through a 1 mm sieve. Thus, the fractions 0.25–1 mm and >1 mm were obtained. The solid material separated into particle size fractions was dried, the masses of fractions 0.002–0.01, 0.01–0.05, 0.05–0.1, 0.1–0.25, 0.25–1, and >1 mm were measured, and the mass proportions were calculated.

For the analysis, three broad particle size fractions of solid material were considered (based on classification of soils by particle size)^43,44^: coarse sand and gravel (referred to as the coarse fraction) (>1 mm), fine sand (0.1–1 mm), and dust (0.002–0.1 mm).

### Mineralogical analysis

Mineral analysis was conducted on particles in the size fraction 0.05–0.1 mm. The samples were powdered in a jasper mortar and analysed by X-ray diffraction (XRD) using a Shimadzu XRD-7000 powder X-ray diffractometer with Сu Kα radiation (λ = 1.5406 Å; 40 kV power and 30.0 mA current). The XRD patterns were obtained with a 1° min^−1^ step across the angular range of 3–70°. The preliminary qualitative phase analysis of the samples was carried out by the main reflections using the Powder Diffraction File-2+ database^45^. To perform the quantitative full profile analysis, the diffractograms were analysed using the Rietveld method with SiroQuant software^46,47^.

Anthropogenic particles were selected from the particle size fraction 0.25–1 mm by visual diagnostic methods taking into account particle shape, morphology, structure, shine, colour, hardness, elasticity, and density. Each particle was photographed with an optical microscope. Particle chemical composition and structure were determined with a scanning electron microscope (SEM; JSM-6390LV, Japanese Electron Optics Laboratory) combined with energy dispersive X-ray spectroscopy (INCAEnergy 350 X-Max 50, Oxford Instruments).

### Chemical analysis

Total concentrations of elements in the granulometric size subsamples were determined with inductively coupled plasma mass spectrometry (ELAN 9000; Perkin Elmer Inc., USA). Solid samples were prepared by extraction with three acids (HNO_3_, HClO_4_, and HF). Sample preparation and analysis procedures were performed according to the technique for measuring metal concentration in solid samples by spectrometry with inductively coupled plasma^48^, which is similar to the US EPA method^49^. Quality control for these measurements was assured through use of certified methodologies and accreditation of the Chemical Analytical Centre of the Institute of Industrial Ecology by The Russian System of the State Accreditation Laboratories.

## Results

The study was carried out at six sampling sites located in various parts of Ekaterinburg. Based on the results of site surveys, a generalized scheme of the sampling sites is presented in Figure 1. Each site consisted of a facade area (the part of the street adjacent to the residential block) and an intra-yard area (courtyard). The facade area included roads, parking lots, sidewalks, and lawns adjacent to the street. The courtyard area included intra-yard driveways (passages), sidewalks, lawns, playgrounds, and sport grounds. The average area of sampling sites was approximately 5,500 m^2^, of which intra-yard space accounted for two-thirds. Parking lots and driveways occupied 37% of the courtyard area (see Table S1 in Supplementary information). The green areas with highly projecting shrub-herb cover in summer, including lawns, children’s playgrounds and sports grounds, covered an average of approximately 60% of the intra-yard space. Driveways and sidewalks had impervious asphalt surfaces. The facade areas were usually equipped with surface water drains that received urban runoff.

According to the results of the survey conducted in February 2017, SDS formed on roads, driveways, and sidewalks. If snow was not removed just after snowfall, SDS was observed on surfaces where cars or pedestrians had passed. The more solid matter present in SDS, the greyer the colour of the dirty snow was. During snow removal, SDS from roads, driveways, and sidewalks was ploughed and stored in snow piles on roadsides and adjacent lawns (Figure 1). In the case of a long period without snow removal accompanied by daytime thaws, a compressed layer of SDS and ice formed on sidewalk and driveway surfaces. When removed from these surfaces, this material was also stored in snow piles. When a layer of ice and compressed snow appeared on roads, the road cleaning service used deicing agents. The melted mass that formed after the use of deicing agents was also stored on curbsides and in piles before transfer to a snow dump area. On sidewalks, a fine gravel (>1 mm) was used for deicing purposes.

Samples of the SDS were collected from road and driveway surfaces. In total, 9 samples of snow and 21 samples of SDS were collected. If SDS was taken from a pile, the sample was assigned to the landscape zone from which the material was transferred to this pile. Samples of undisturbed snow cover were taken from areas located at significant distances (>10 m) from roads and driveways.

The solid phase material content in samples of undisturbed snow cover was <1 g L^−1^ of meltwater. A considerably higher average solid phase content, approximately 35 g L^−1^, was observed in SDS samples collected from roads and sidewalks (Figure 2).

The particle size composition from a granulometric analysis of SDS solid phase material is presented in Figure 2 for the four landscape zones. On sidewalks, 78% of the total solid phase mass was associated with the coarse fraction with size >1 mm. Gravel particles in the SDS solid phase on sidewalks represented deicing material. The coarse fraction was also observed in other landscape zones and was associated with the transfer of material from sidewalks.

The dust fraction predominated on roads, where it contributed two-thirds of the total solid phase mass and three-quarters in all landscape zones when the coarse fraction was not considered. Among the dust fractions, the 0.05–0.1 mm fraction made the largest contribution to the SDS solid phase mass. The contribution of the 0.002–0.01 mm fraction was approximately 10% of the solid matter. The size distribution of solid particles <1 mm was roughly the same in all landscape zones. The total solid phase material contents in SDS samples collected from lawns and driveways were 6.6 and 12 g L^−1^ of meltwater, respectively and these values were significantly lower than those of samples collected from the roads.

**Figure 3:**
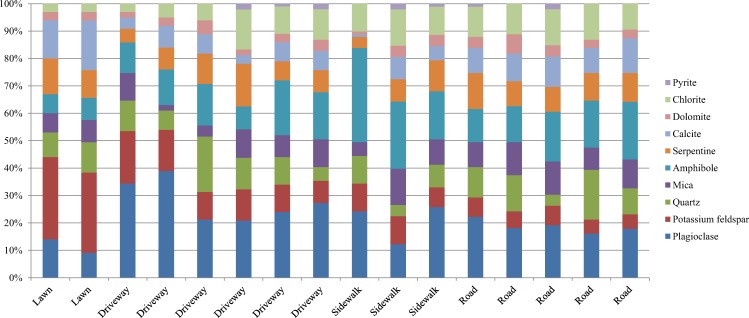
Mineral composition of the solid phase in 16 SDS samples with a particle size of 0.05–0.1 mm.

A mineral analysis of 16 SDS solid phase samples with particle sizes of 0.05–0.1 mm is presented in Figure 3. In all samples, the mineral composition was composed of plagioclase, potassium feldspar, quartz, mica, amphibole, serpentine, calcite, dolomite, and chlorite, and in some samples, pyrite was found. Despite the samples coming from different geographical parts of the city located at significant distance from each other (3–12 km) and from different geological units, their mineral composition was fairly uniform, especially for SDS samples from the roads.

The minerals found in SDS samples were attributed to three main groups: felsic rocks, mafic rocks, and building materials. Felsic rocks are represented by granite, which may include all the detected potassium feldspar and portions of the plagioclase, quartz, and mica. Some of the plagioclase and mica, as well as all the amphibole, serpentine, and chlorite, likely came from mafic rocks (gabbro, serpentinite, and hornblendite). Building materials contain calcite and dolomite. The minerals attributed to building materials were likely weathering and abrasion products of asphalt concrete, concrete, and plastered surfaces.

**Figure 4:**
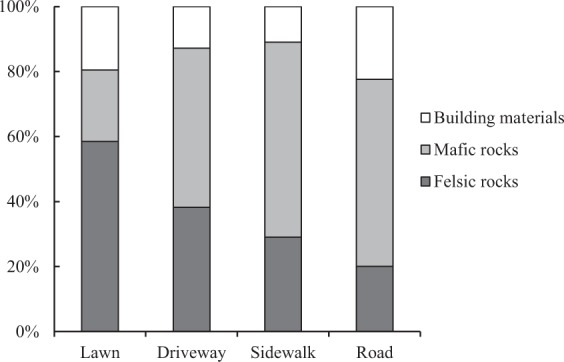
Mineral composition of the solid phase in the SDS.

The mineral composition of the SDS solid phase according to the three main groups of rocks and materials is presented in Figure 4. The contributions of felsic and mafic rocks varied with landscape zone. Felsic rocks formed the bulk solid phase in two SDS samples taken from lawns. The proportion of felsic rocks decreased in the order lawn–driveway–sidewalk–road. More than 50% of SDS solid material from sidewalks and roads in the facade area was sourced from mafic rocks. The largest contribution of weathering and abrasion products of building materials to the 0.05–0.1 mm solid phase SDS fraction was found in samples taken from roads (approximately 4 g L^−1^ of meltwater, which was >20% of the total mass). In general, the main differences in SDS mineral composition among landscape zones were associated with different contributions of felsic and mafic rocks.

A mineralogical analysis carried out on the coarse fraction of anti-icing agents sampled from a sidewalk showed the presence of amphibole and chlorite. Thus, the anti-icing fine gravel used on sidewalks was derived from the mafic rocks.

**Figure 5:**
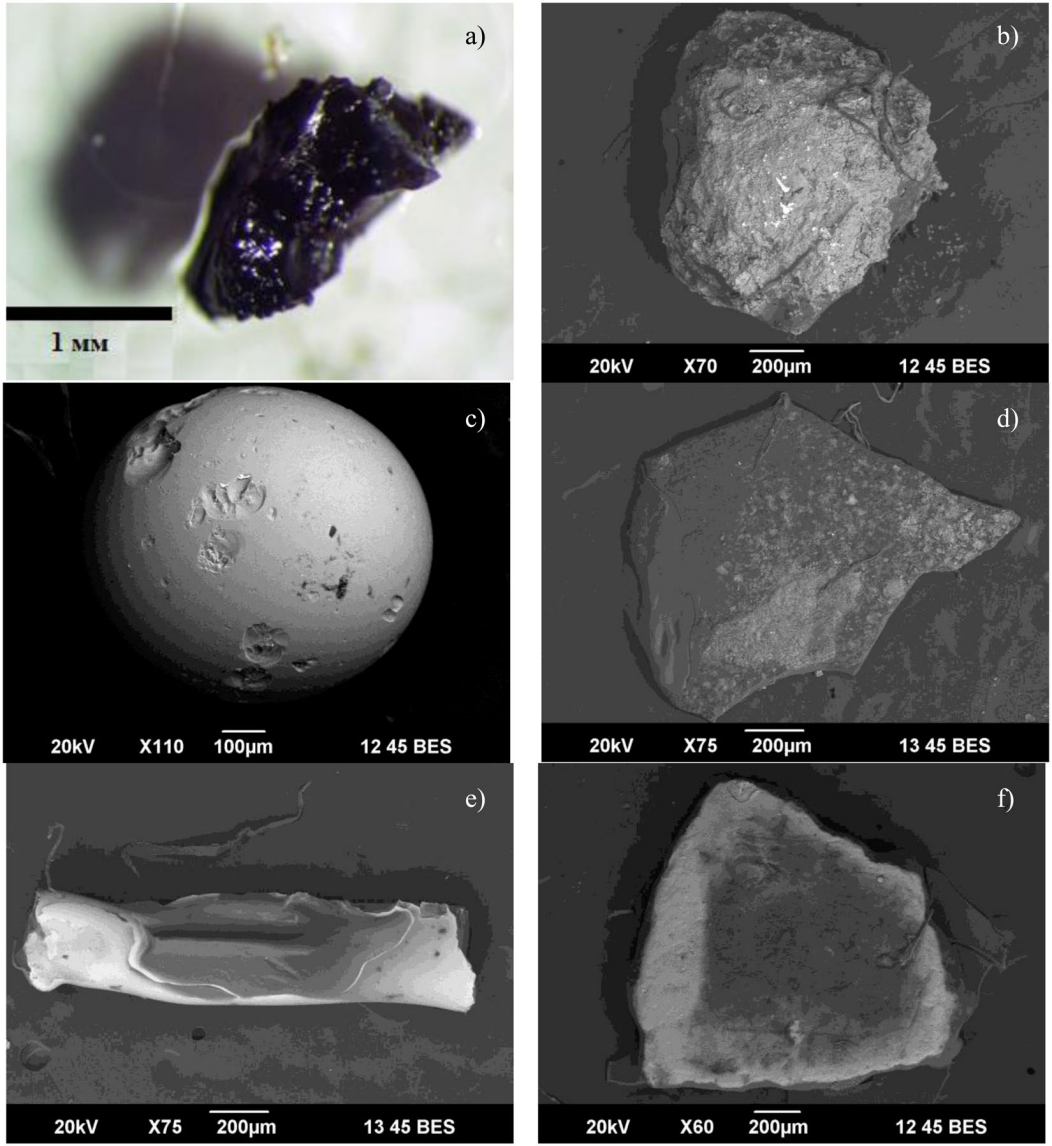
Examples of anthropogenic particles found in the solid phase of SDS. Microscope picture: (**a**) granulated slag particle. SEM images: (**b**) stone-like slag particle; (**c**) silicate microsphere; (**d**) piece of foil; (**e**) tyre fragment; (**f**) plaster fragment.

Technogenic particles in SDS were represented by granulated and lithoid slag, silicate microspheres, foil, and car tyre and plaster fragments (Figure 5). The granulated slag particles are glassy angular semi-transparent formations characterized by сonchoidal fracture. Silicon oxide dominated (up to 49%) the composition of this particle type. Ca, Mg, and Fe oxides were found in smaller quantities. Impurities of Al oxide, and more rarely V, were also observed. The CaO to SiO_2_ ratio was less than 1.5, and therefore, such slag particles were attributed to acid slags^50^. The lithoid slag was represented by porous devitrified particles with heterogeneous chemical composition close to that of the granulated slag. Oxides of Si (up to 42%) and Fe (up to 20%) prevailed. Ca, V, and Mg oxides were found in lesser quantities, and these particles were also classified as acid slags. Insignificant impurities of K, Na, Al, and Ti oxides were noted. Silicate microspheres or aluminosilicate hollow microspheres form during high-temperature flaring of coal and other high ash content fuels. The particle size depends on the presence of iron oxides in their composition. The silicate microspheres in this study were characterized by the predominance of Si oxide (>70%). Silicate-ferruginous microspheres contained iron oxide (up to 18–20%) and Ca, Al, Mg, and Na oxide impurities. A high Ca oxide content and low levels of Si and Mg oxides characterized the chemical composition of the plaster fragments, as is typical for cement and concrete.

**Figure 6:**
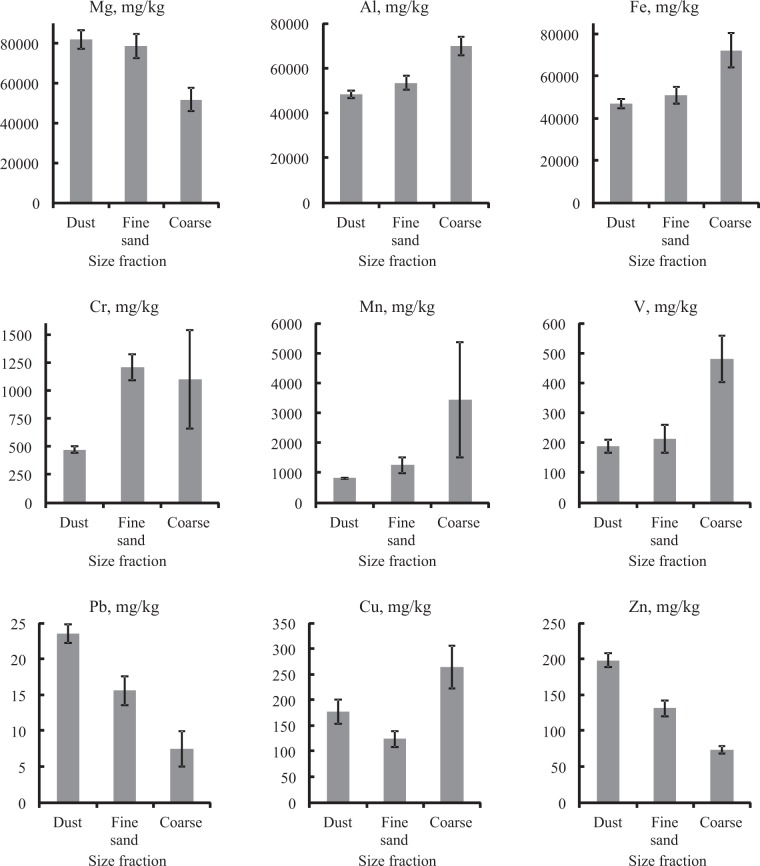
Average concentrations of metals in the solid phase of SDS by size fractions.

The average metal concentrations in the large particle size fractions of the SDS solid phase from the samples are presented in Figure 6 for the typomorphic major elements Mg, Al, and Fe, the typomorphic minor elements Cr, Mn, and V, and the suggested anthropogenic origin elements Pb, Cu, and Zn. The figure shows the association of Al, Fe, Mn, V, and Cu contents with the coarse fraction. The relationship between the concentration of typomorphic major elements (Mg, Al, and Fe) and particle size fraction may indicate differences in the mineral content of the coarse fraction from the dust and fine sand fractions. The dust and fine sand fractions of the SDS had the highest concentration of Mg and the lowest concentrations of Al, Fe, Mn, and V. There were no differences in concentrations of these elements in the two smallest particle size fractions (i.e., dust and fine sand).

The elevated Cr concentrations in the fine sand and coarse fractions were associated with abrasion of the sieve material used to separate the fractions in the granulometric analysis. The sieves consisted of steel alloys containing Fe and Cr in a ratio of 8:1 and Fe, Cr, and Ni in a ratio of 8:2:1. Steel flakes several microns in diameter with the same ratio of Fe, Cr, and Ni were detected by SEM analysis on the surfaces of particles selected from these size fractions.

**Figure 7:**
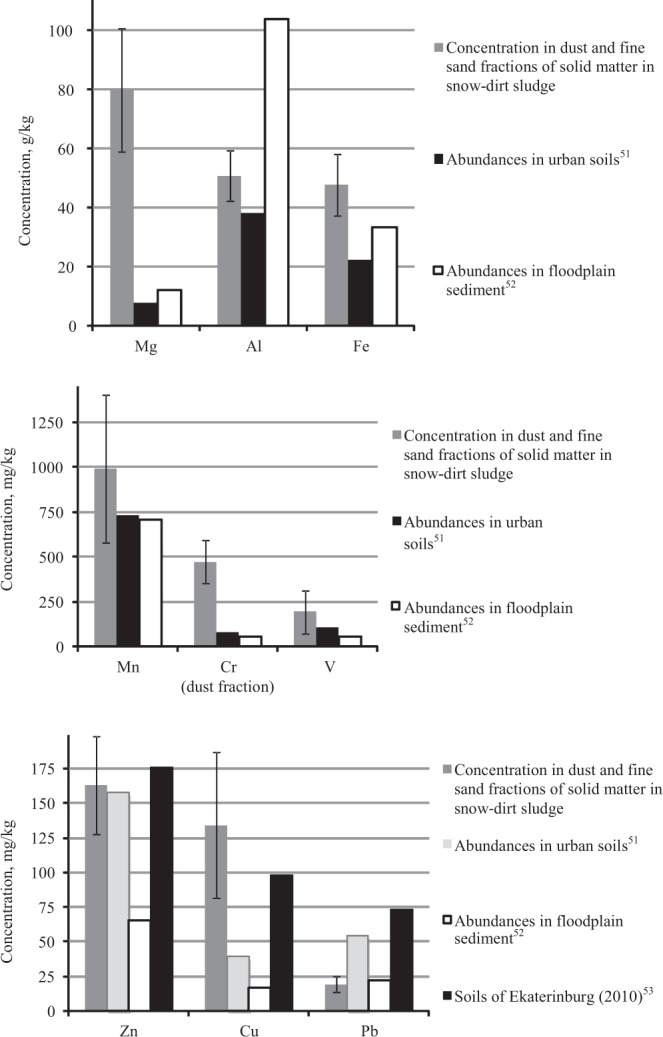
Comparison of the geochemical spectra of the SDS solid phase for nine metals with data on the abundance of these metals in urban soils^51,53^ (average total element concentrations in samples from the upper 30 cm soil horizon, without granulometric separation of the samples) and sediments of river floodplains of Europe^52^ (average total element concentrations, sampling depth 0–25 cm, <2 mm grain size sediment). The concentration of Cr is represented by the dust fraction of solid matter in snow-dirt sludge.

A comparison of the geochemical associations was conducted among the SDS solid matter obtained in Ekaterinburg and the abundances of metals in urban soils^50^ (average total concentration of the element in samples from the upper 30 cm soil horizon; granulometric analysis of the studied soil samples was not carried out) and in sediments from river floodplains^52^ (representing average total element concentrations of sediment with a grain size of <2 mm from a sampling depth of 0–25 cm) (Figure 7). The average concentrations in the dust and fine sand fractions are presented in Figure 7, which shows that the geochemical association of the SDS solid phase in Ekaterinburg clearly differs from the other geochemical associations. Compared with urban soils, the SDS had a significant excess of typomorphic major and minor elements (Mg, Al, Fe, Cr, and V). A comparison of average PHE (Zn, Cu, and Pb) concentrations in Ekaterinburg soil obtained from the results of a 2010 soil survey^53^ is also presented.

As seen in Figure 7, the Zn concentration in the SDS solid phase and in urban soils was the same. Considerable differences were found between the average Pb concentrations in SDS and urban soils. The Pb concentration in contemporary surface sediment found in the SDS was more than 2.5 times lower than the abundance in urban soils^51^ and almost 4 times lower than in Ekaterinburg soil in 2010^53^. The current Pb concentration in the solid phase of the SDS was close to the values observed in the river floodplain sediment. The concentration of Cu in both the SDS solid material and Ekaterinburg soil were much higher than that in urban soils^51^.

## Discussion

The urban environmental compartment that forms on roads and sidewalks in winter as a result of the mixing of snow and USDS through the actions of cars and pedestrians is defined in this article as SDS. Studying the formation of SDS and the conservation of surface runoff in winter allows for a more detailed and comprehensive analysis of both sedimentation and environmental processes in urban areas.

The mass of the solid material in SDS may depend on the intensity of USDS mixing with snow, as well as on the thickness of the deposited sediment. In the framework of this study, the dependence on mixing intensity is reflected in the significant differences in solid sediment concentrations per litre of meltwater in samples taken on sidewalks, in driveways, and on roads. The average content of solid sediment in the SDS on roads with heavy traffic was approximately 35 g L^−1^ of meltwater, which was considerably higher than the content of solid matter in samples of undisturbed snow. The dust fraction made up the largest portion of SDS solid sediment. The fraction <0.01 mm was also present in SDS in significant quantities, exceeding the precipitation of particulate matter from the atmosphere, which is characterized by the content of solid mater in undisturbed snow.

Taking into account the depth of snow cover (35.1 cm) and water accumulation in it (103.8 L m^−2^) made it possible to calculate the amount of solid phase SDS that accumulated in Ekaterinburg. A 23 g L^−1^ dust fraction concentration in meltwater corresponded to a solid phase SDS surface density of approximately 2.4 kg m^−2^ on roads, and the total mass of the dust fraction mixed with snow on roads was approximately 80,000 tons. This value reflects the amount of road-deposited sediments preserved by the snow cover for the winter period. The mass of the SDS dust fraction associated with roads in the city was up to 53 kg per capita. The total quantity of USDS was even greater due to deposition of sediments over other surfaces. Thus, USDS is a significant source of suspended particulate pollution to the atmosphere, storm sewers, and river systems.

The mineral composition of the SDS solid phase generally reflected the geology of Ekaterinburg and the surrounding area, which is composed of a mosaic of felsic and mafic complexes^54^. These rocks form deposits that are used for building materials. The SDS samples collected in various parts of the city were quite homogeneous in their solid matter mineral composition and were not fully classifiable as either felsic or mafic rocks. The proportion of these two types of rock varied depending on the function of the landscape where the samples were collected. In zones with the highest anthropogenic impact, such as roads, the main solid matter contribution to SDS was made by mafic rocks. Almost all SDS samples contained a relatively large amount of debris and fragments from calcium carbonate-based building materials, as indicated by the calcite and dolomite signatures in the mineral analysis. The presence of calcium-containing materials and carbonates is a general characteristic of the urban environment^55^. Apparently, the fraction of the solid sediment with a size >1 mm on the sidewalks was completely associated with amphibole, serpentinite, and other types of gravel used in a deicing mix on the sidewalks. The presence of anthropogenic particles in the SDS confirms the significant anthropogenic impacts on urban landscapes. Slag particles, including spherules that are characteristic of high-temperature technological processes, were mainly found in Ekaterinburg.

The SDS solid phase chemical composition contained major elements specific to the minerals and materials that constitute the solid sediment, specifically containing compounds of Mg, Al, Fe, and Ca used in building materials. Differences in the concentrations of Mn, V, and Cu in the granulometric fractions were apparently due to the addition of gravel particles that did not belong to USDS itself. The absence of variability in the metal content between the dust and fine sand fractions indicated a common genesis for these particles, as did the lack of enrichment with these elements during the grinding of the coarse fraction. It can be assumed that small particles resulted from the destruction of larger particles. Confirmatory evidence was provided by the higher dust fraction proportion on roads where road deposited sediment was subject to intense physical impact.

There was significant variability in the concentrations of Pb, Cu, and Zn in the granulometric fractions. The anthropogenic contribution of these elements can be quite large in urban environments. Higher levels of these metals in the dust fraction may be associated with adsorption, which occurs more intensively on particles with higher specific surface areas. The distribution of pollutants in the granulometric fractions reflects the processes of pollution intake and redistribution in the urban environment.

Lead concentrations in the SDS solid phase were relatively low. The average Pb concentration in the dust fraction did not exceed 25 mg kg^−1^, whereas in urban soils, the concentration was 2–3 times greater according to observations over the previous decade^6,7^. The Pb content may have decreased as a result of the 1997 ban on leaded gasoline. The low Pb content in contemporary samples thus suggests that USDS is an indicator of environmental processes. The high Cu and Zn contents in the SDS solid sediment and urban soils provide evidence of current pollution associated with these metals, the source of which is likely traffic emissions.

When assessing the health risk of SDS, it is necessary to consider both the large dust fraction and PHE concentration. High contents of fine sediment containing high pollutant concentrations are found in places where SDS is stored during the spring snowmelt runoff event. The solid matter in SDS is partially deposited in depressed zones and forms polluted puddles in the urban landscape^6,7^. When the puddles dry, deflation can occur, leading to the suspension of particulate matter with sizes of <0.01 mm, which can reach human breathing height. Fractions <0.1 mm can be transferred through the sewage system and surface waterways into rivers and lakes where it will contribute to bottom sediments.

In the urban sedimentary cascade model, the top of the cascade contains the products of soil erosion and abrasion of roads and other surfaces, and the subsequent stages include temporary deposition or storage prior to further transport, as in many depositional systems^3,4^. For territories with a long period of low temperatures and snow cover, the urban sediment cascade model should take into account this seasonal factor. In the winter, the formation of SDS on roads, driveways, and sidewalks preserves surface runoff, which is then the main driver of lateral USDS transport for a period of months. Conservation of surface runoff changes the accumulation regime and the dynamics of the sedimentation process. In many cities, snow removal, snow storage in piles, and snowmelt are important and understudied factors in the sedimentation process.

Having data on the size fractions and mineral composition of the SDS solid phase in Ekaterinburg allows speculation that the abrasion of road surfaces by studded car wheels is a primary source of sediment in Ekaterinburg in the winter period. The high contribution of mafic rocks to the sedimentary material deposited on roads is associated with the gravel used in road construction and provides evidence of roadway abrasion. In the studied region, the use of granite deposits in building construction was banned in the early 1990s due to the high content of naturally occurring radionuclides^56^. Therefore, the available materials made of mafic rocks are extensively used in construction, including road construction. Road surface abrasion with studded wheels leads to the formation of fine dust. Intensive car traffic leads to a uniform distribution of solid sediment throughout the city. The mineralogical, chemical, and granulometric properties of SDS sampled from roads were the same in all geographic areas of the city.

Parking is a problem in Ekaterinburg, as well as in other cities with extensive automobile utilization. The landscape survey of the sampling sites found that approximately one in five cars was parked outside of arranged lots (red coloured items in Figure 1), particularly on lawns. This manner of parking destroys the soil surface layer and curbs. As a result, illegitimate parking facilitates an additional source of erosion material involved in sedimentation.

If no current source of pollution deposits Pb into road sediments, it is assumed that the Pb is supplied to the sediment by erosion of soils contaminated with Pb before the ban on leaded gasoline. The low Pb content in contemporary sediment may indicate a low Pb concentration in the surface layer of soils and, at the same time, intense soil erosion in the urban environment.

Detailed *in situ* descriptions with specially designed landscape surveys are an important tool for contemporary urban sedimentology. The description of sampling sites characterized vehicle-related areas (e.g., roads, driveways, and parking areas) on which SDS is formed. Quantitative assessment of traffic characteristics revealed the coherence of individual landscape zones within the urban sediment cascade. The potential of landscape surveys in urban sedimentological and geochemical studies is even greater in the summer when there is no snow cover.

There is likely a common origin for USDS and the solid phase found in SDS. To obtain the necessary evidence for comparative analysis, the granulometric, mineral, and chemical composition of winter SDS and USDS sampled in the summer period would need to be conducted. Such a study will provide additional data regarding seasonal features of sedimentation in the urban environment.

The sedimentation processes in winter, the formation of SDS, and the intensive melting of sludge with a high fine solid particle content are important factors influencing the quality of the urban environment. The seasonal characteristics of urban sedimentation require the attention of municipal and regulatory bodies in regions with a long winter period of low temperatures and snow cover. To reduce the harmful effects of solid sediment accumulation in the snow cover, the solid sediment content in the snow in different land use areas and landscape zones requires control. When planning measures to remove snow from the streets, differences in SDS formation in different parts of the urban landscape and the intense mud runoff during the period of snowmelt in residential areas and on the roads also require consideration. SDS removal and transport outside of residential areas will reduce both surface runoff during snow melt and the total mass of accumulated USDS. Special measures are needed for solid sediment treatment in places where SDS is stored so that accumulation of fine solid material, deflation in the dry period, and sediment transfer into water bodies are prevented. When high levels of solid sediment accumulate, SDS should be considered along with other municipal wastes, and appropriate treatment processes for this material should be implemented.

## Supplementary information

Supplementary information
