# Supplementary material for: Snow-dirt sludge as an indicator of environmental and sedimentation processes in the urban environment
Source: Sci Rep. 2019 Nov 21;9:17241. doi: 10.1038/s41598-019-53793-z (PMC6872592; doi:10.1038/s41598-019-53793-z)
Supplement: Supplementary file 1 — Supplementary information [file 41598_2019_53793_MOESM1_ESM.pdf]

Supplementary information

Title: Snow-dirt sludge as an indicator of environmental and sedimentation processes in the urban environment

Authors:

Andrian Seleznev, Ilia Yarmoshenko, Georgy Malinovsky, Ekaterina Ilgasheva, Elena Baglaeva, Anastasia Ryanskaya, Daria Kiseleva, Tamara Gulyaeva

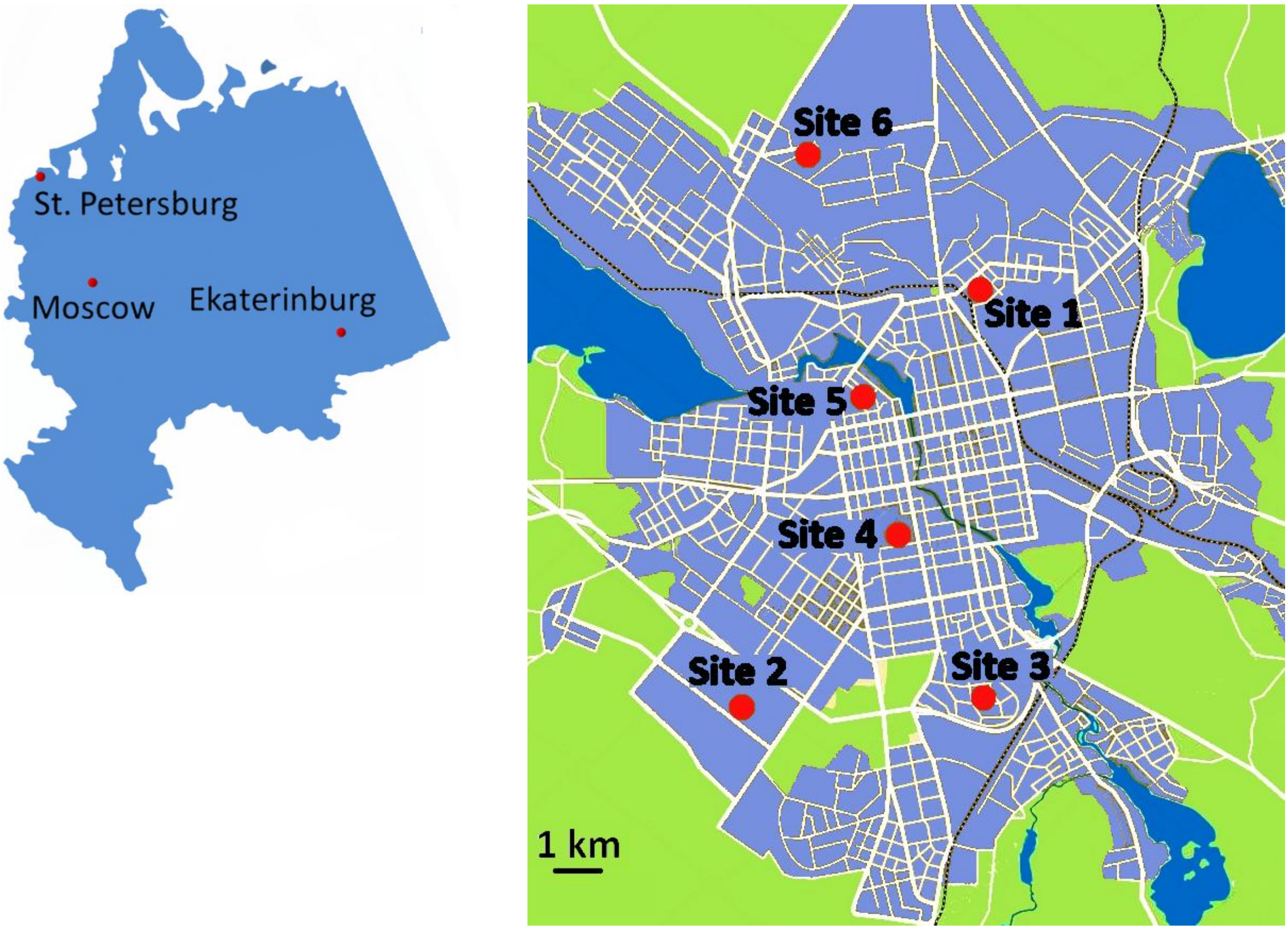

Fig 1S. Maps of Russia and Ekaterinburg with locations of sampling sites.

Table S1. Main characteristics of the sampling sites.

Site, street, coordinates	Year of development	Total area*, m^2	Area of roads, %	Area of driveways, %	Area of sidewalks, %	Area of lawns, %	Number of parking lots
Site 1, Smazchikov Str., 56.8595 N / 60.6228 E	1969	5,900	14%	28%	4%	55%	32
Site 2, Krasnolesye Str., 56.7813 N / 60.5440 E	1991, 1993	5,700	16%	28%	11%	46%	67
Site 3, Rodonitovaya Str., 56.7931 N / 60.6193 E	1997	4,400	5%	23%	9%	63%	50
Site 4, Frunze Str., 56.8133 N / 60.6052 E	1973	7,400	16%	20%	6%	58%	67
Site 5, Zhukova Str., 56.8391 N / 60.5886 E	1980, 1993	5,500	12%	34%	9%	45%	120
Site 5, Mashinostroiteley Str., 56.8832 N / 60.5684 E	1963, 1988	4,300	5%	29%	3%	63%	49

Without building structures

Table S2. Number of undisturbed snow and SDS samples.

Microlandscape functional zones	Undisturbed snow	SDS*
lawn	9 (0)	2 (2)
sidewalk	0	6 (3)
driveway	0	8 (6)
road	0	5 (5)

*The number of samples subjected to chemical and mineralogical analysis is shown in parenthesis.

Examples of snow and SDS sampling in the urban environment are presented in Fig 2S–7S.

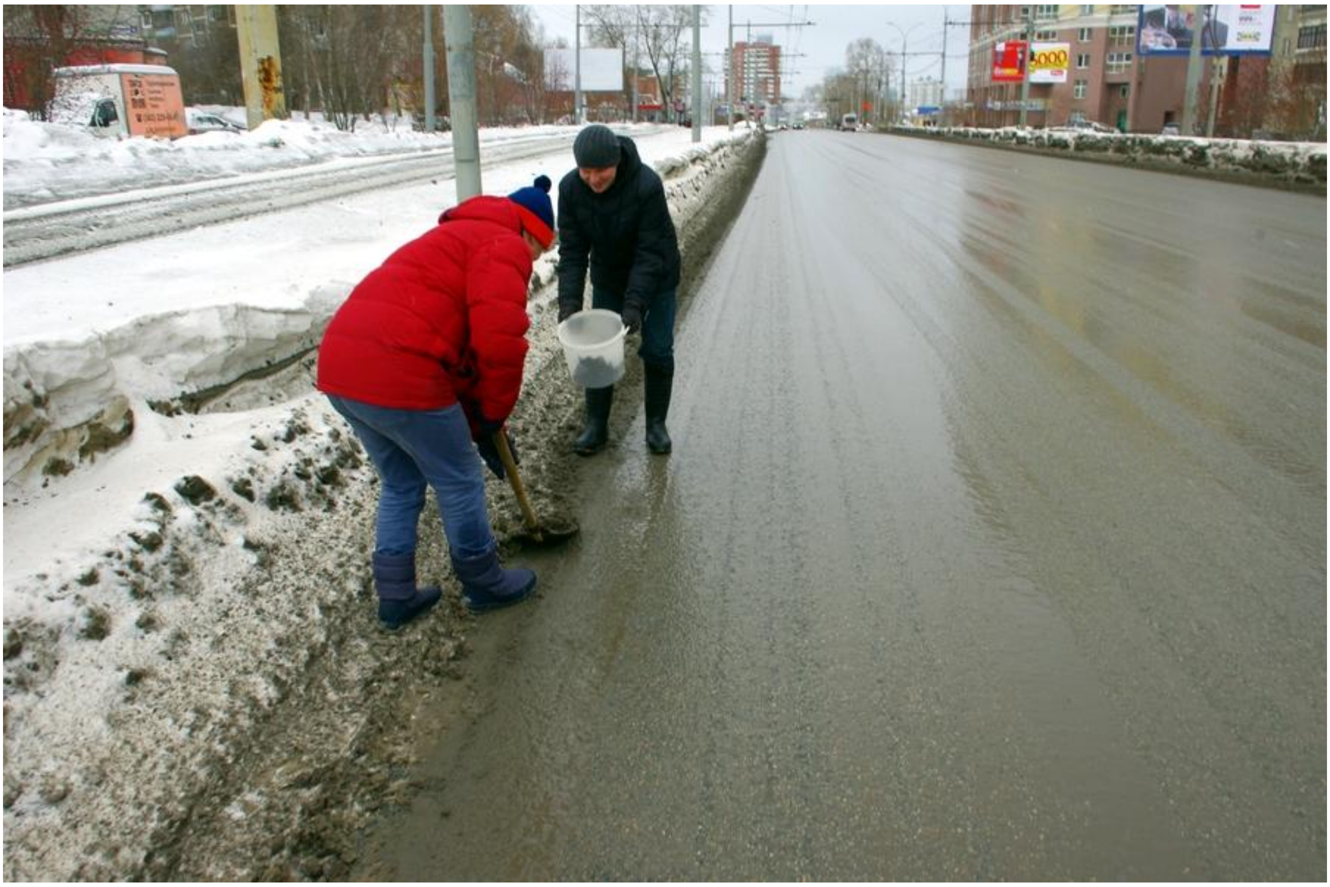

Fig. 2S. SDS sampling on the road.

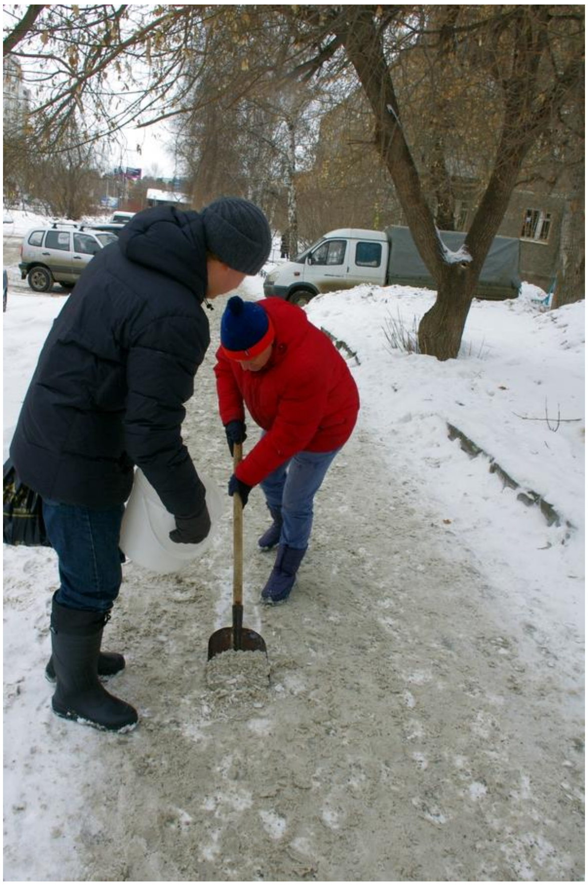

Fig. 3S. SDS sampling at sidewalk.

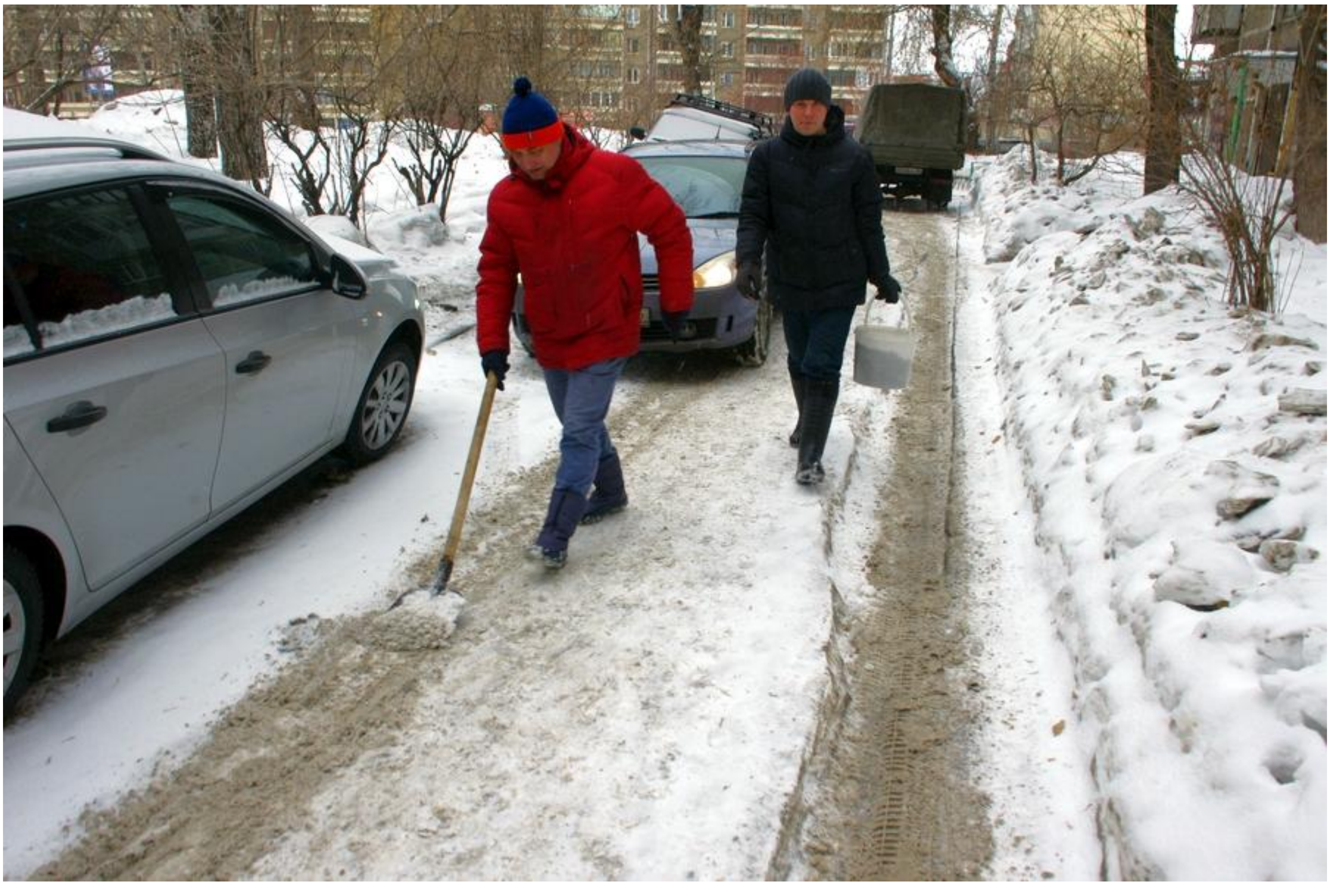

Fig. 4S. SDS sampling at driveway.

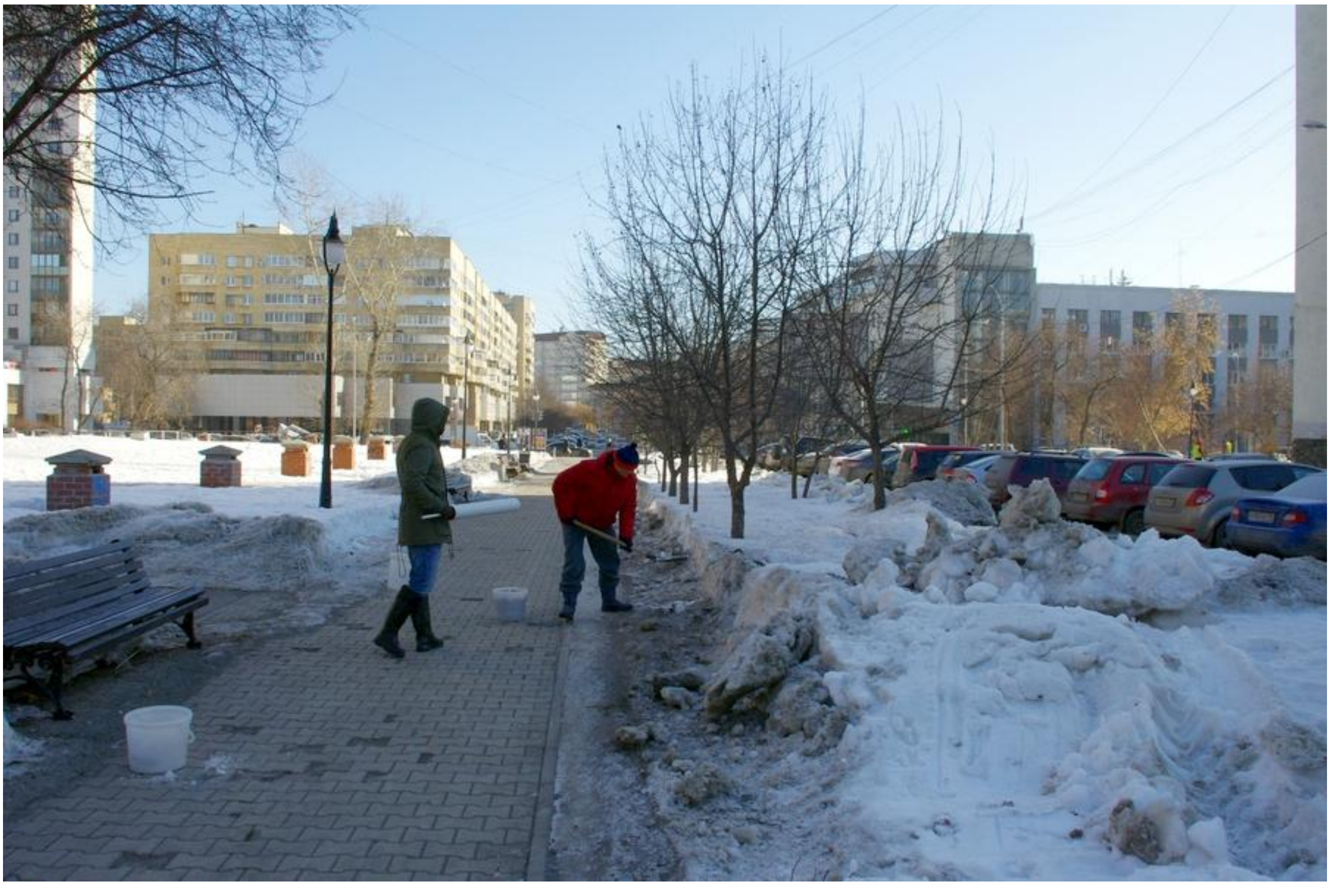

Fig. 5S. SDS sampling at lawn.

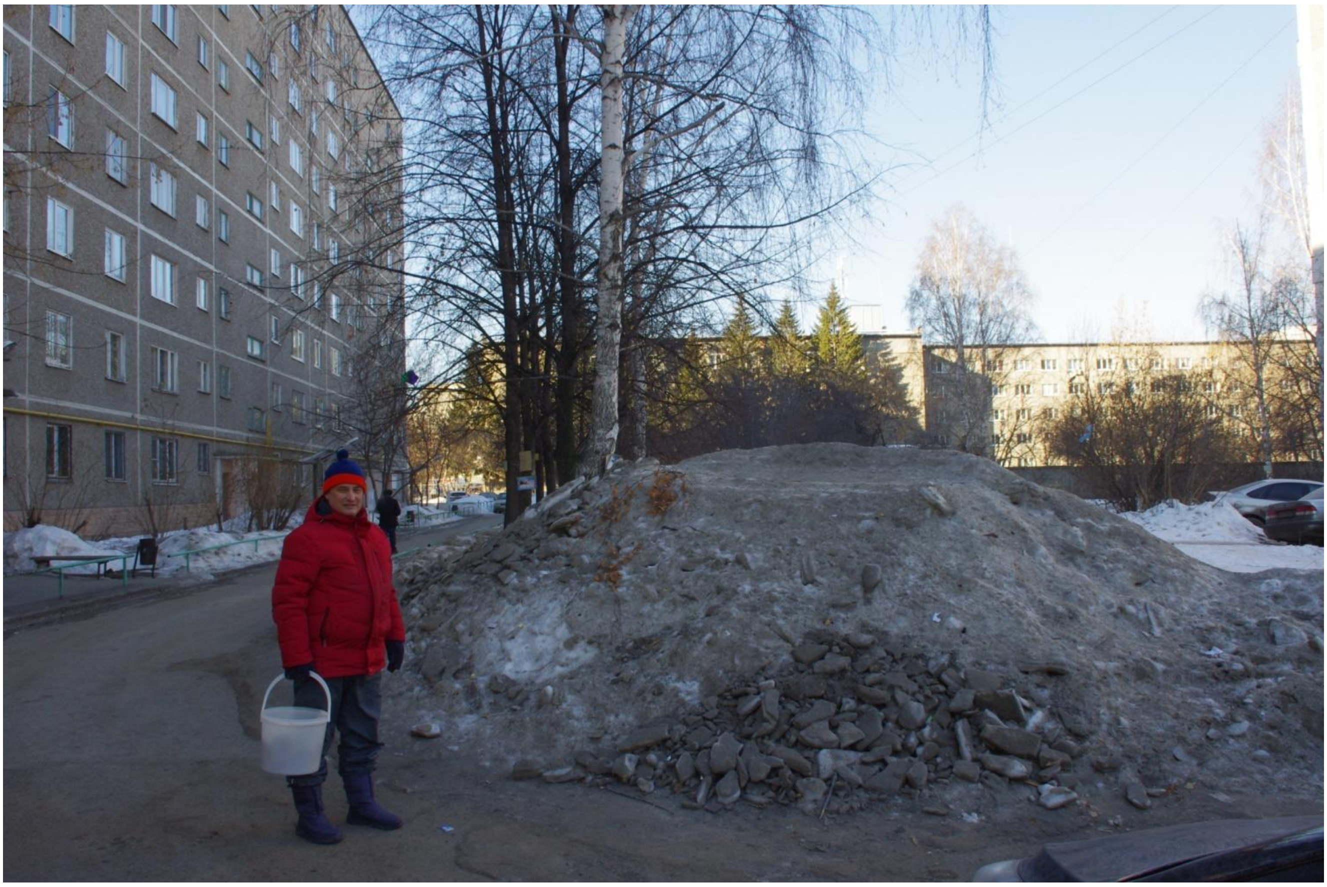

Fig. 6S. SDS sampling from snow pile.

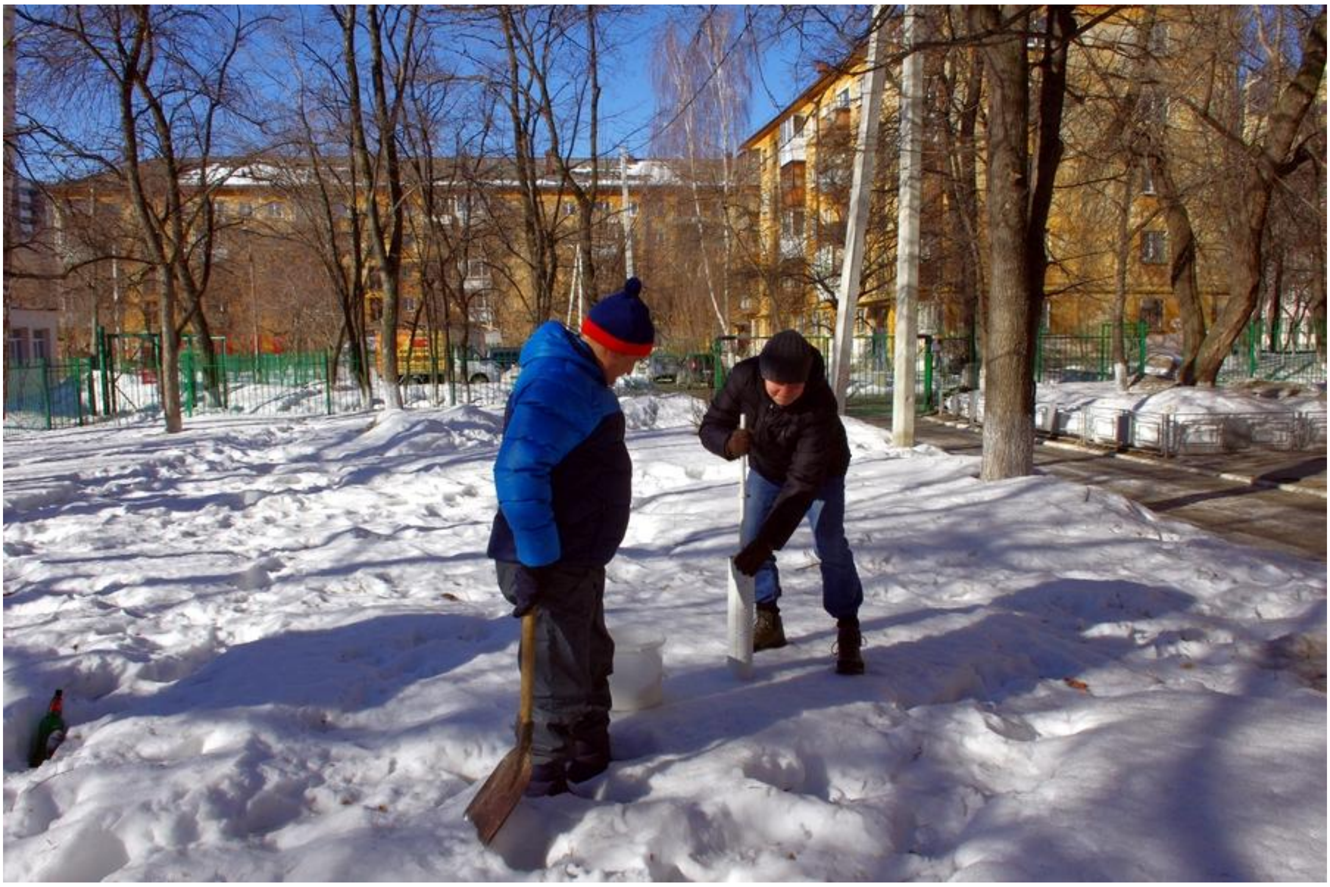

Fig. 7S. Undisturbed snow sampling.
